# Graphene Oxide, a Novel Nanomaterial as Soil Water Retention Agent, Dramatically Enhances Drought Stress Tolerance in Soybean Plants

**DOI:** 10.3389/fpls.2022.810905

**Published:** 2022-02-15

**Authors:** Lin Zhao, Wei Wang, Xiaohong Fu, An Liu, Jinfeng Cao, Jianfeng Liu

**Affiliations:** ^1^School of Life Sciences, Institute of Life Science and Green Development, Hebei University, Baoding, China; ^2^Hebei Key Laboratory of Crop Salt-Alkali Stress Tolerance Evaluation and Genetic Improvement, Cangzhou, China; ^3^Hebei Research Center for Geoanalysis, Baoding, China

**Keywords:** soybean, graphene oxide, drought stress, defense enzyme, drought-related hormones, drought-related genes

## Abstract

Drought is one of the most severe environmental stressors that place major constraints on the growth of soybeans (*Glycine max* L.). Graphene oxide (GO) is a nanomaterial that can promote plant growth without toxic effects. In this study, the physiological and molecular responses to drought stress with GO treatment were examined. We discovered that the relative water content (RWC) of stems and leaves treated with GO was 127 and 128% higher than that of the WT plants, respectively. The root parameters in GO-treated soybeans were increased by 33, 38, 34, and 35% than WT plants in total root length, root surface area, root diameter, and root volume, respectively. The activities of superoxide dismutase (SOD), catalase (CAT), peroxidase (POD), and ascorbate peroxidase (APX) were also increased by 29, 57, 28, and 66%, respectively. However, the relative conductivity (REC), malondialdehyde (MDA), and hydrogen peroxide (H_2_O_2_) accumulation were remarkably decreased. Furthermore, the content of drought-related hormones JA, SA, and ABA in GO-treated soybeans increased by 32, 34, and 67% than WT plants, respectively. At the molecular level, the effects of GO treatment were manifested by relatively higher expression of four drought-related genes: *GmP5CS*, *GmGOLS*, *GmDREB1*, and *GmNCED1*. Taken together, our findings revealed that GO could directly increase plant defense enzymes, hormone content, and the expression of drought-related genes, thereby improving the soybean’s ability to resist drought. These findings could provide new opportunities for improving drought tolerance in soybeans through effective soil water retention agents.

## Introduction

Soybean (*Glycine max* L.) is one of the most important foods and oil crops. In addition to being a rich source of nutrition and minerals, soybeans also contain secondary metabolites such as isophorone (Shadakshari et al., 2011), saponins, phytic acid, oligosaccharides, thyroid hormones (Jo’keefe et al., 2015), and phytoestrogens (Ososki and Kennelly, 2003). However, soybean roots require more water during their growth and development stage to avoid getting undeveloped. Therefore, drought stress is a major constraint to soybeans production and yield stability (Pan et al., 2011). Soybean yields are reduced by about 40% every year due to drought (Yu et al., 2008). Recently, genetic engineering for drought tolerance with candidate genes has been reported in the major food crops, and efforts for developing drought-resistant soybean lines are in progress (Wang, 2014). However, this approach is time-consuming and expensive because drought-resistant genes need to be cloned and transformed into soybean plants to verify drought tolerance. Finding effective techniques to improve soybean drought tolerance and water usage efficiency is critical for increasing soybean yield.

As a new type of nanomaterial, graphene has been widely used in many fields because of its beneficial physical and chemical properties. Graphene oxide (GO) is one of the most important graphene derivatives because it contains many oxygen-containing hydrophilic functional groups that can be modified, such as carboxyl, carbonyl, epoxy, and hydroxyl groups (Nazari et al., 2020). GO has a greater surface activity, better biocompatibility, and is simpler to construct and functionalize than graphene. As a result, graphene oxide has received a lot of attention since its discovery in 2004 (Hu et al., 2018). GO is now used in many aspects of our life, particularly in biological fields such as biosensor development, photothermal treatment, and medication delivery (Chong et al., 2014). The use of GO in agricultural production, particularly in promoting plant growth and development, has been reported in recent years.

Previous studies showed that different concentrations of GO have different biological effects on plants, ranging from growth stimulation to acute toxicity. For example, treatment with 500–2,000 mg/L GO reduced seed germination, and 0.1–10 mg/L significantly increased arsenic phytotoxicity in wheat (*Triticum aestivum* L.) (Hu et al., 2014; Vochita et al., 2019). Moreover, treatment with 25–100 mg/L GO inhibited seminal root growth in *Brassica napus* L. and significantly increased ABA content (Cheng et al., 2016). All these studies have proved that GO treatment can inhibit plant growth and lead to crop stress. However, studies have shown that GO positively impacts plant growth, such as treatment with 20 mg/L GO promoted tomato (*Lycopersicon esculentum* Mill.) growth through the ABA and IAA pathways (Jiao et al., 2016). Exposure with 0.1 mg/L GO could be used as an antibacterial agent to extend the vase life of *Rosa chinensis* Jacq. (He et al., 2018b), and 50 mg/L GO promoted the germination of *Spinacia oleracea* L. and *Allium schoenoprasum* L. in the soil (He et al., 2018a). However, relatively few studies have investigated the effect of GO on drought tolerance. Recently, it has been found that GO as an effective soil water retention agent can confer drought stress tolerance to *Paeonia ostii* without causing toxicity (Zhao et al., 2020). Furthermore, the effects of GO nanosheets were in a drought context, with the osmotic and antioxidant protection conferred by GO nanosheets to drought shoots, leading to higher shoot biomass (Lopes et al., 2021). The findings indicated that GO might increase the drought tolerance of woody plants, but no research on the drought tolerance of soybean plants treated with GO has yet been undertaken.

In this study, we focused on the impact of GO on physiological and biochemical traits linked to drought tolerance mechanisms in soybeans under drought stress, and aimed at explaining the mechanism of GO improving drought tolerance of plants from the aspects of water content, root growth state, membrane stability, stomatal control, plant hormone and gene expression. These findings will provide new opportunities for improving drought tolerance in soybeans through effective soil water retention agents. Although the focus of this research is on soybeans, we find it likely that the underlying concepts can be used to improving drought tolerance in other crops as well.

## Materials and Methods

### Plant Cultivation and Treatments

**FIGURE 1:**
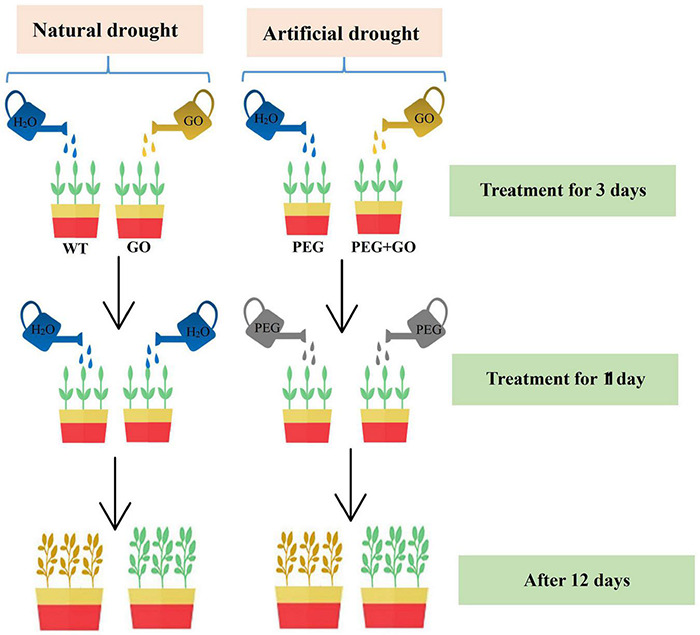
The schematic illustrating was laid out in our experiment.

The Cangdou 09-Y1 was provided by Cangzhou Academy of Agriculture and Forestry Sciences. The growth chamber conditions include a day/night temperature of 28/20°C, 75% relative humidity, and a 16 h (5:00–21:00) photoperiod provided by fluorescent light (240 μEm^–2^ s^–1^). To study growth conditions that artificial drought and natural drought, the soybean plants with 6–7 true leaves was divided into four groups of 24 pots per group, with non-GO treated (WT) and GO groups under natural drought. Both the polyethylene glycol (PEG) and PEG + GO groups were defined as simulated drought. The different treatments for the four groups are as follows (Figure 1):

WT: Each plant was watered daily for 3 days with 100 mL of water, thereafter with 50 mL of water for 1 day, and then they were not watered.
GO: Each plant was watered daily for 3 days with 100 mL of 100 μg/mL GO solution, thereafter with 50 mL of water for 1 day, and then they were kept in a waterless state.
PEG: Each plant was watered daily for 3 days with 100 mL of water, then 50 mL 15% PEG-6000 solution was treated once to achieve drought stress.
PEG + GO: Each plant was watered daily for 3 days with 100 mL of 100 μg/mL GO solution, then 50 mL 15% PEG-6000 solution was treated once to achieve drought stress.

Finally, samples were taken at 0, 6, and 12 days after treatments to determine the various traits and anatomical structures.

### Determination of Relative Water Content

Three pots of 0.2 g roots, stems and leaves were taken from each treatment. The relative water content of the roots, stems and leaves was determined using the saturation weighing method proposed by Wang et al. (2014). Three pots of 10 g soil were taken from each treatment. The soil water content was measured using the soil drying weighing method described by Wang et al. (2014).

### Evaluations of Root Morphology

To evaluate the effect of GO on soybean roots under drought stress, the root morphological parameters of three pots of soybean in each treatment were analyzed by root scanner (Seiko Epson Corp., Tokyo, Japan). The total length, surface area, and volume of the roots were measured using WinRHIZO 4.0b software.

### Analysis of Physiological Indexes Related to Drought Tolerance

Diaminobenzidine (DAB) staining was used to detect the accumulation of hydrogen peroxide (H_2_O_2_) (Watanabe and Lam, 2008). The leaves were completely immersed in DAB staining solution and treated at 25°C for 2 h under the light. 70% ethanol was added after thoroughly sucking out the DAB, washing four times with distilled water, and eliminating the dye solution adsorbed on the leaves. The mixture was decolorized by boiling for 10 min in a water bath to see the dyed leaves (Zhang et al., 2010).

The conductivity was measured using a DDBJ-350 conductivity meter (Liu et al., 1997). In addition, H_2_O_2_ and malondialdehyde (MDA) contents were detected with a kit (Nanjing Jiancheng Bioengineering Co., Ltd., China). The free proline (Pro) content was determined by a colorimetric method using a kit (Nanjing Jiancheng Bioengineering Co., Ltd., China).

### Determination of the Defense-Related Enzymes

We placed leaf samples (0.1 g) in a precooled mortar and added 0.5 ml precooled phosphate buffer for grinding to obtain tissue homogenate. Then, the tissue homogenate was centrifuged at 10,000 rpm at 4°C for 10 min, and the supernatant was taken to determine antioxidant enzyme activity. The activities of four antioxidant enzymes—superoxide dismutase (SOD), peroxidase (POD), catalase (CAT), and ascorbate peroxidase (APX)—were determined using reagent kits (Nanjing Jiancheng Bioengineering Co., Ltd., China).

### Assessments of Chlorophyll Content and Chlorophyll Fluorescence Parameters

We measured the chlorophyll content at 8:30 a.m. using a portable chlorophyll meter (SPAD-502Plus, China). Subsequently, the chlorophyll fluorescence parameters were measured using a chlorophyll fluorescence spectrometer (Heinz Walz GmbH 91090, Germany) after the plants stood for 2 h in the dark. This system recorded minimal fluorescence from the dark-adapted leaf (Fo) and non-photochemical quenching coefficient (qN). The photochemical quenching coefficient (qP) and the actual photosynthetic efficiency of photosystem II [Y(II)] were calculated. Moreover, we also calculated the maximum quantum efficiency of photosystem II (PSII) photochemistry [variable fluorescence from the dark-adapted leaf (Fv)/maximum fluorescence from dark-adapted leaf (Fm)]. All the parameters were measured on the top leaves of 6 different plants in one group on the same day.

### Observation and Determination of the Anatomical Structures

We used nail polish to evaluate the stomatal morphology of the lower epidermis of soybean leaves. We tore off pieces of leaves to temporarily load the lower epidermis of the soybean leaves. We coated the back of the leaves with transparent nail polish, let it air-dry, tore off epidermal cells with tweezers, spread them on a glass slide, dropped a drop of clear water, and tabulated them for observation.

Cross-sections of roots and leaves were made by the paraffin section. We cleaned the test material, cut the root from the middle, and cut small squares of 1 cm × 1 cm from both sides of the major vein of the leaf. All samples were put into FAA fixing solution (mixed with 70% ethanol, glacial acetic acid, and formaldehyde at a volume ratio of 18:1:1) for fixation. We then made permanent slices with a thickness of 8–12 μm by the conventional paraffin slicing method (Miao et al., 2014).

The anatomical structures of roots and leaves were photographed using an Olympus BX53 biomicroscope. TopView software was used to measure the anatomical parameters of leaves, including the thickness of the root epidermis, cortex, endodermis, xylem and phloem. The thickness of the thickness of the upper epidermis, lower epidermis, palisade tissue, and spongy tissue were also measured.

### Gene Expression Analysis

Quantitative real-time PCR (qRT-PCR) was used to detect the expression levels of genes linked with drought tolerance. RNA extraction was performed following the manufacturer’s instructions of the RNA Kit (DNase I) (CW2598S, CWBIO). The cDNA was synthesized using RNA as a template and the UEIris II RT-PCR system for First-Strand cDNA Synthesis (with dsDNase) kit (R2028, US Everbright ^®^Inc., Suzhou, China). The ACTIN gene in soybean plants was used as the internal reference gene using a 2 × Fast Super EvaGreen qPCR Master Mix kit.

The thermal cycling program was as follows: 95°C for 5 min, followed by 45 cycles of 95°C for 15 s and 58°C for 60 s. ACTIN gene was used as a reference gene to normalize target gene-expression levels in the soybean samples. The relative expression levels of genes was calculated by the 2^–△△CT^ method with three independent biological replicates. The qRT-PCR analysis was repeated in triplicate. All primers are listed in Supplementary Table 1.

### Extraction and Determination of Hormone Levels

The ABA, IAA, JA, and SA of soybean were determined by Beijing BioDee Biotechnology Co. Ltd., Beijing, China, and the methods were modified from those described by Pan et al. (2002). Approximately 0.5 g dehulled grains were ground in a pre-cooled mortar that contained 5 mL extraction buffer composed of isopropanol/hydrochloric acid. The extract was shaken at 4°C for 30 min. Then, 10 mL dichloromethane was added, and the sample was shaken at 4°C for 30 min and centrifuged at 13,000 rpm for 5 min at the same temperature. We then extracted the lower, organic phase. The organic phase was dried under N_2_ and dissolved in 150 μL methanol (0.1% methane acid) and filtered with a 0.22 μm filter membrane. The purified product was then subjected to high-performance liquid chromatography-tandem mass spectrometry (HPLC-MS/MS) analysis. HPLC analysis was performed using a ZORBAX SB-C18 (Agilent Technologies) column (2.1 mm × 150 mm; 3.5 mm). The mobile phase A solvents consisted of methanol/0.1% methanoic acid, and the mobile phase B solvents consisted of ultrapure water/0.1% methanoic acid. The injection volume was 2 μL. MS conditions were as follows: the spray voltage was 4,500 V; the pressure of the air curtain, nebulizer, and aux gas were 15, 65, and 70 psi, respectively; and the atomizing temperature was 400°C.

### Statistical Analysis

The relative expression of genes and physiological index is shown as means ± standard deviations (SD) from three independent experiments. Treatments results were compared by a two-sample *t*-test using Origin software (version 8.5). *P* < 0.05 or *P* < 0.01 were supposed to be significant for all tests.

## Results

### Graphene Oxide Enhanced Drought Tolerance of Soybean Plants

**FIGURE 2:**
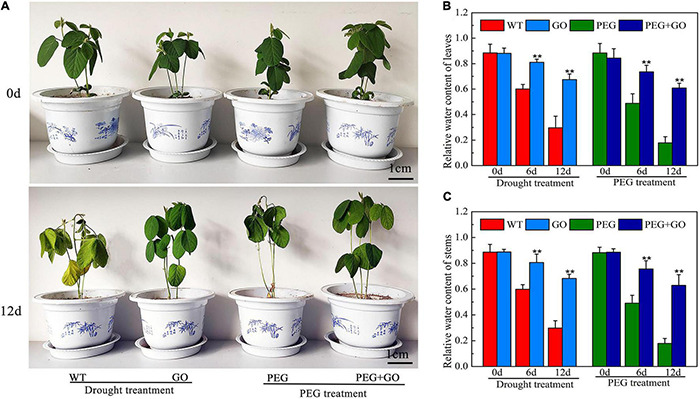
Effects of GO on growth performance of soybean under drought and PEG stress. **(A)** Phenotype of soybean. **(B)** The RWC of the leaves. **(C)** The RWC of the steams. Values in the figure represent the means ± SD of three technical replicates. Student’s *t*-test for pair comparison in each treatment: **P < 0.01.

To analyze the effect of GO on plant growth, we treated the soybean plants with simulated drought and natural drought, respectively. The growth performance of soybean seedlings was first observed under both natural and simulated drought stress. No difference was observed in the growth performance of seedlings before GO treatment. The WT and PEG-treated plants had the drought-sensitive symptom of wilting leaves, but the soybean leaves treated with GO showed no obvious changes under drought for 12 days (Figure 2A and Supplementary Figure 1). In addition, the relative water content (RWC) of leaves and stems treated with GO was 127% ± 0.1 and 128% ± 0.25 higher than that of the WT plants under natural drought for 12 days, respectively. The RWC of leaves and stems treated with GO was 241% ± 0.65 and 253% ± 0.24 higher than the plants treated with PEG for 12 days, respectively (Figures 2B,C). These results showed that GO could be used to enhance the drought tolerance of soybean plants.

### Graphene Oxide Improved the Root Parameters of Soybean Plants

**FIGURE 3:**
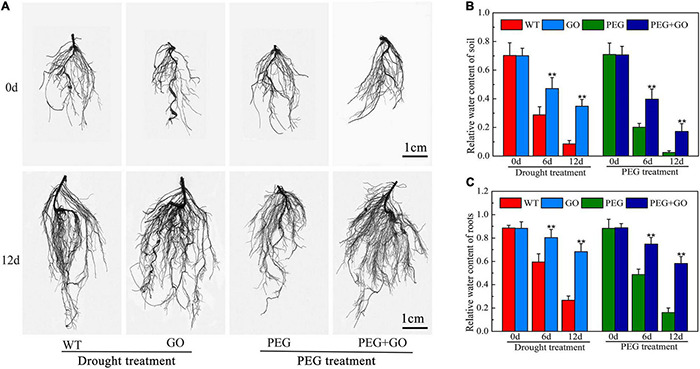
Effects of GO on root system of soybean plants under drought and PEG stress. **(A)** Images of soybean root systems scanned. **(B)** RWC of soil potted soybeans. **(C)** RWC of soybean roots. Values in the figure represent the means ± SD of three technical replicates. Student’s *t*-test for pair comparison in each treatment: **P < 0.01.

Developing an adequate root system is critical for plants to take up sufficient water at low soil moisture. We found that the growth performance of roots was similar among all groups before both drought treatments. However, the roots of GO-treated plants showed significantly stronger performance, with denser root systems under both types of drought stress for 12 days (Figure 3A and Supplementary Figure 1). Compared with WT plants, the total root length, root surface area, root diameter, and root volume of GO-treated plants increased by 33.41% ± 0.07, 37.88% ± 0.06, 34.25% ± 0.08, and 34.72%, respectively, under natural drought stress for 12 days (Table 1). We also found that the variation trend of root parameters under simulated drought was consistent with those under natural drought conditions. Compared with PEG plants, the total root length, root surface area, root diameter, and root volume of GO-treated plants increased by 44.26% ± 0.07, 46.64% ± 0.11, 26.19% ± 0.05, and 50.00% ± 0.13 under simulated drought (Table 1). Results showed that the RWC in the roots and soil of potted soybeans was not substantially different before the GO treatment. However, the RWC of GO-treated roots and soil was 156% ± 0.28 and 220% ± 0.45 higher than those of WT plants under natural drought for 12 days, respectively. Moreover, there were similar results under simulated drought: the RWC increased in GO-treated roots and soil by 262% ± 0.27 and 562% ± 0.34 compared with those in the PEG-treated plants, respectively (Figures 3B,C). Therefore, GO treatment could prevent soil moisture evaporation, leading to enhanced root water absorption capacity and further promoting root growth of soybean plants.

**TABLE 1:** Root parameters of soybeans under drought stress.

Time	Treatments	Root length (m)	Root surface (mm^2^)	Root diameter (mm)	Root volume (mm^3^)
0d	WT	2.23 ± 0.66	2,943 ± 407	4.0 ± 0.3	310 ± 100
	GO	2.22 ± 0.77	2,909 ± 479	3.9 ± 0.5	330 ± 40
	PEG	2.3 ± 0.14	2,810 ± 432	4.1 ± 0.2	320 ± 110
	PEG + GO	2.20 ± 0.14	2,706 ± 460	4.0 ± 0.5	310 ± 30
12d	WT	6.30 ± 0.36	8,452 ± 1,720	4.3 ± 0.3	910 ± 220
	GO	8.39 ± 1.96**	1,1345 ± 2,167*	5.9* ± 1.4	1,220 ± 180**
	PEG	6.43 ± 1.19	7,727 ± 1,271	4.2 ± 1.0	740 ± 130
	PEG + GO	9.28 ± 2.30**	11,330 ± 1,745**	5.3 ± 1.3*	1,110 ± 90**

Values in the figure represent the means ± SD of three technical replicates. Student’s t-test for pair comparison in each treatment are *P < 0.05, **P < 0.01.

### Graphene Oxide Improved Soybean Reactive Oxygen Species System

**FIGURE 4:**
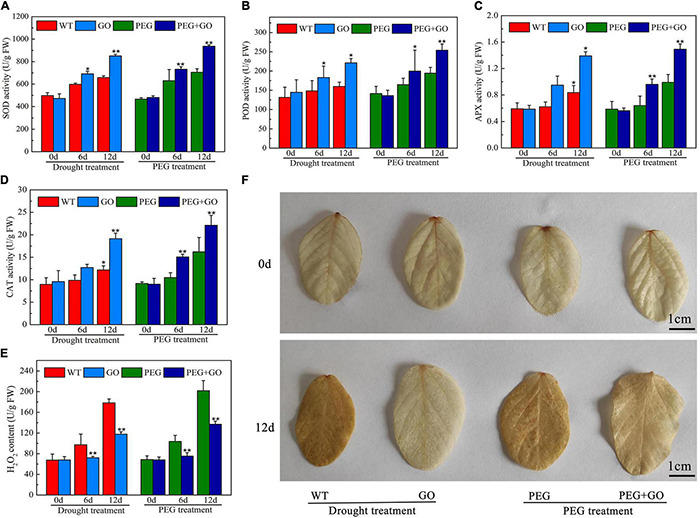
Defense enzyme content of soybean with GO treatment under drought and PEG stress. **(A)** SOD content. **(B)** POD content. **(C)** APX content. **(D)** CAT content. **(E)** H_2_O_2_ content. **(F)** H_2_O_2_ accumulation was detected by DAB staining. Values in the figure represent the means ± SD of three technical replicates. Student’s *t*-test for pair comparison in each treatment: *P < 0.05, **P < 0.01.

The SOD, CAT, POD, and APX may efficiently eliminate reactive oxygen species (ROS) and free radicals from plants, regulate plasma membrane peroxidation and prevent drought damage to membrane structures. We discovered that before both types of drought stress, the contents of SOD, POD, and APX in each group were consistent, however, after 12 days of natural drought stress, SOD, POD, and CAT activities increased in GO-treated leaves by 29.16% ± 0.05, 27.67% ± 0.09, and 66.43% ± 0.24 compared with those in WT soybeans, respectively (Figures 4A–C). In addition, SOD, POD, and CAT activities was 32.81% ± 0.05, 32.68% ± 0.03, and 38.56% ± 0.04 higher than those of PEG-treated plants under natural drought for 12 days (Figures 4A–C). The CAT content was increased, while the H_2_O_2_ content decreased with GO treatment (Figures 4D,E). Further, we visually observed H_2_O_2_ accumulation in leaves with DAB staining, and there was no significant difference before GO treatment. However H_2_O_2_ accumulation is inhibited in GO-treated leaves under both natural and simulated drought stress (Figure 4F), which is consistent with the detection results of hydrogen peroxide content in Figure 4E.

### Graphene Oxide Regulated the Osmotic Substances

**FIGURE 5:**
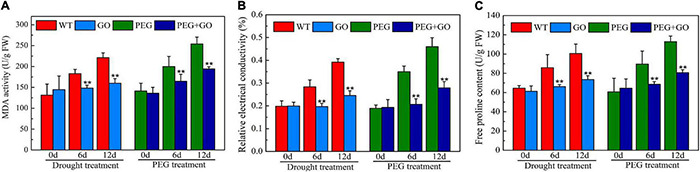
Content of osmotic adjustment substances in soybean leaves under drought and PEG stress. **(A)** MDA content. **(B)** Relative electrical conductivity (REC). **(C)** Free proline content (Pro). Values in the figure represent the means ± SD of three technical replicates. Student’s *t*-test for pair comparison in each treatment: **P < 0.01.

The REC and MDA are crucial indicators of cell membrane damage. The REC and MDA contents constantly increased with the extension of natural drought stress duration, while the contents of REC and MDA in GO treatment decreased by 37% ± 0.07 and 28% ± 0.09, respectively, compared with WT under 12 days of drought treatment (Figures 5A,B). The REC and MDA content also decreased by 39% ± 0.03 and 24% ± 0.06 after PEG treatment for 12 days (Figures 5A,B). In addition, the content of Pro decreased by 27% ± 0.05 and 28% ± 0.07 compared with those in non-GO leaves under both treatment after 12 days, respectively (Figure 5C).

### Graphene Oxide Treatment Leads to Chlorophyll Parameters Changes

**FIGURE 6:**
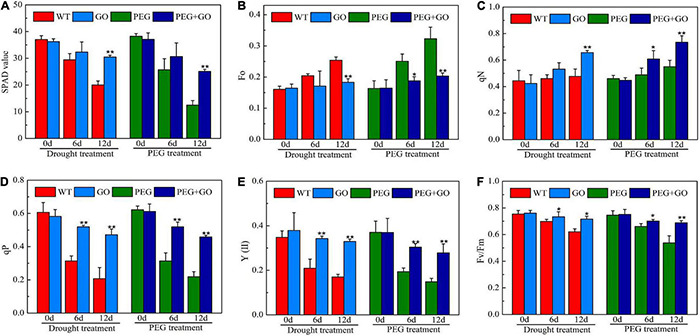
Chlorophyll parameter in soybean leaves under drought and PEG stress. **(A)** SPAD values. **(B)** Minimum fluorescence (Fo). **(C)** Non-photochemical quenching coefficient (qN). **(D)** Photochemical quenching coefficient (qP). **(E)** The actual photosynthetic efficiency of photosystem II [Y(II)]. **(F)** The maximum quantum efficiency of photosystem II (PSII) photochemistry (Fv/FM). Values in the figure represent the means ± SD of three technical replicates. Student’s *t*-test for pair comparison in each treatment: *P < 0.05, **P < 0.01.

The SPAD value is a useful index for determining a plant’s relative chlorophyll concentration. In our study, the soybeans’ SPAD value showed a downward trend when the drought stress duration was increased. However, the SPAD value was significantly higher by 52% ± 0.11 and 101% ± 0.27 than non-GO soybeans under both drought treatments, respectively (Figure 6A). qN was higher, while Fo was lower in GO-treated soybean plants (Figures 6B,C). The qN and Fo values were significantly increased by 27.82% ± 0.04 and 36.45% ± 0.08, respectively, under natural drought stress. Similarly, the qN and Fo values were significantly higher than the PEG group by 58.85% ± 0.11 and 33.49% ± 0.03, respectively, under simulated drought stress (Figures 6B,C). In addition, qP, Fv/Fm, and Y(II) exhibited downward trends when exposed to natural drought. The qP, Fv/Fm, and Y(II) increased by 104% ± 0.23, 88% ± 0.09, and 15% ± 0.01, respectively, under natural drought for 12 days. Fv/FM and Y(II) were 127, 94, and 28% higher than PEG plants under simulated drought for 12 days, respectively (Figures 6D–F). As a result, GO may protect the reaction center during drought stress by reducing the capture of light energy and the electron transfer efficiency through photosystem II.

### Graphene Oxide Affected Anatomic Structure of Soybean Leaves

**FIGURE 7:**
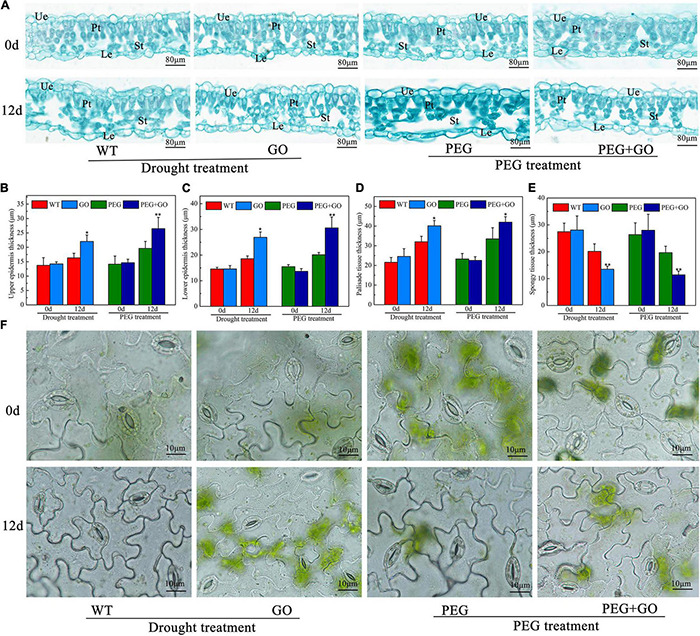
Anatomic structure analysis of soybean leaves under drought and PEG stress. **(A)** Anatomical images of the soybean leaves. **(B)** Thickness of upper epidermis of soybean leaves. **(C)** Thickness of lower epidermis of soybean leaves. **(D)** Thickness of palisade tissue of soybean leaves. **(E)** Thickness of spongy tissue of soybean leaves. **(F)** Stomata in the lower epidermis of soybean leaves. Ue, Upper epidermis; Le, Lower epidermis; Pt, Palisade tissue; St, Spongy tissue. Values in the figure represent the means ± SD of three technical replicates. Student’s *t*-test for pair comparison in each treatment: *P < 0.05, **P < 0.01.

The anatomical structures of soybean leaves were examined, and the leaf structure parameters were further quantified to investigate the effect of GO treatment on leaves’ anatomical structures under drought stress. We found that the palisade and spongy mesophylls in the non-GO-treated group were sparse, and some cells were even destroyed under both types of drought stress (Figure 7A). However, the GO-treated group had thick palisade cells, which were closely arranged (Figure 7A). The upper epidermis, lower epidermis, and palisade tissue of GO-treated plants were significantly increased by 5.69 ± 0.15, 8.34 ± 0.24, and 8.11 ± 0.17 μm, while the spongy mesophyll was significantly decreased by 6.66 μm under natural drought stress. Similarly, the upper epidermis, lower epidermis, and palisade tissue of GO-treated leaves were significantly increased by 6.85 ± 0.13, 10.44 ± 0.26, and 8.47 ± 0.19 μm, respectively, but the spongy mesophyll was significantly decreased by 8.27 μm under simulated drought (Figures 7B–E). No obvious differences were found in stomatal aperture before GO treatment. However, most non-GO-treated stomata were closed in response to drought stresses (Figure 7). Overall, we proved that GO could resist drought by increasing the thickness of the upper epidermis, lower epidermis, and palisade tissue of soybean leaves and decreasing the thickness of spongy mesophyll.

### Graphene Oxide Improved Anatomical Structure of Soybean Root

**FIGURE 8:**
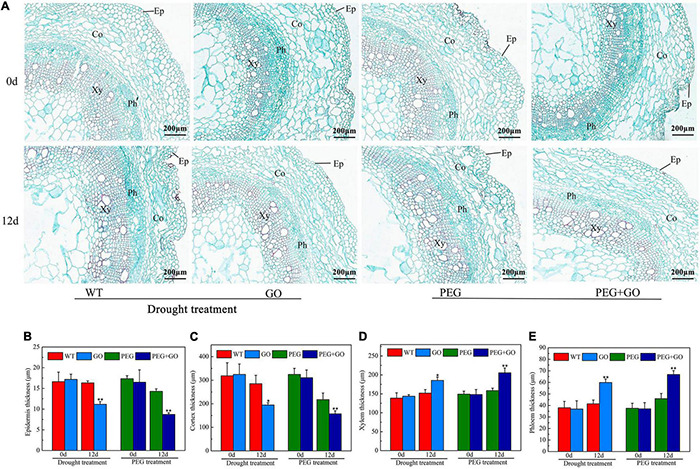
Anatomic structure analysis of soybean roots under drought and PEG stress. **(A)** Anatomical images of soybean roots. **(B)** Thickness of epidermal of soybean roots. **(C)** Thickness of cortex of soybean roots. **(D)** Thickness of xylem of soybean roots. **(E)** Thickness of phloem of soybean roots. Ep, Epidermal; Co, Cortex; Xy, Xylem; Ph, Phloem. Values in the figure represent the means ± SD of three technical replicates. Student’s *t*-test for pair comparison in each treatment: *P < 0.05, **P < 0.01.

The anatomical structure of soybean roots was observed under both types of drought stress. The outer skins of soybean roots were sunken inward, and the parenchyma cells of the inner skin were dehydrated and deformed. The central parenchyma cells were damaged (Figure 8A). However, there was no obvious change in the root anatomy with GO treatment (Figure 8A). The characteristics of the anatomical structures of the roots were also measured. Compared with the WT plant, the thickness of the epidermis and cortex in GO-treated soybean roots decreased by 31.65% ± 0.13 and 31.62% ± 0.07. However, the xylem and phloem thickness increased significantly by 21.91% ± 0.05 and 44.58% ± 0.12 under natural drought. Under simulated drought conditions, the thickness of the epidermis and cortex of GO-treated soybean roots decreased by 64.08% ± 0.23 and 37.81% ± 0.09, respectively, while the thickness of xylem and phloem increased by 29.76% ± 0.07 and 45.53% ± 0.14 (Figures 8B–E). These results showed that GO could increase the thickness of the xylem and phloem by reducing the thickness of the soybean root epidermis and cortex, thereby increasing the water absorption capacity of the root system and resisting drought stress.

### Effects of Graphene Oxide on Drought-Related Hormones

**FIGURE 9:**
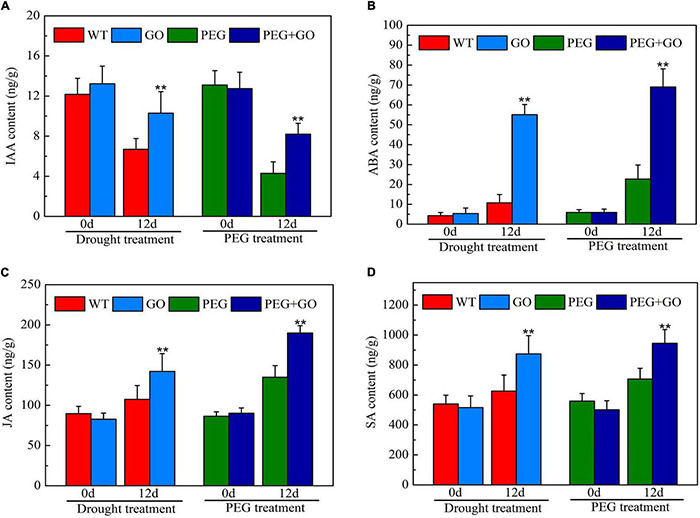
Content of drought-related hormones under drought and PEG stress. **(A)** IAA content. **(B)** ABA content. **(C)** JA content. **(D)** SA content. Values in the figure represent the means ± SD of three technical replicates. Student’s *t*-test for pair comparison in each treatment: **P < 0.01.

The contents of IAA, ABA, SA, and JA were determined to reveal the impacts of exogenous GO on soybean hormones during both types of drought stress. Without the GO treatment, there was no significant difference in IAA, ABA, SA, and JA contents in each group before both types of drought stress (Figure 9). However, the IAA content was significantly increased by 53.96% ± 0.11 and 90.70% ± 0.28 compared with non-GO plants under both types of drought stress (Figure 9A). In addition, the contents of ABA, SA, and JA were significantly increased by 67.10% ± 0.12, 33.68% ± 0.09, and 32.16% ± 0.07 compared with WT plants, respectively, under natural drought. Similarly, ABA, SA, and JA contents were significantly increased by 80.40% ± 0.17, 39.66% ± 0.05, and 40.57% ± 0.10, respectively, compared to the PEG group under simulated drought (Figures 9B–D). Overall, results showed that GO is an activator of IAA, ABA, SA, and JA, promoting plant resistance to drought stress.

### Graphene Oxide Enhanced the Expression of Drought-Resistant Genes

**FIGURE 10:**
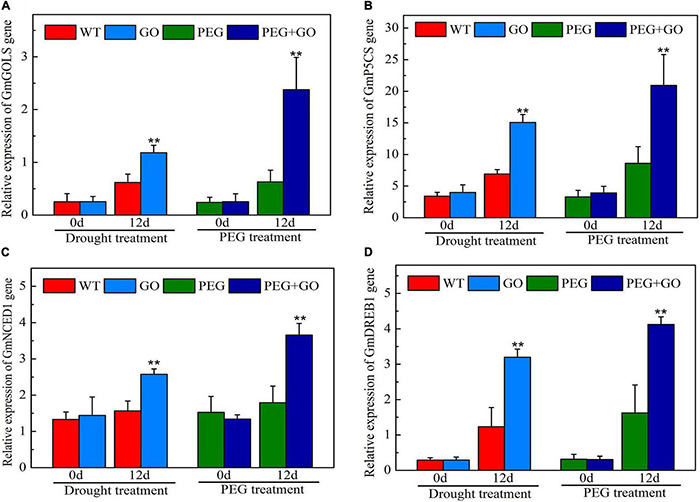
The expression of drought-resistant genes in soybean under drought and PEG stress. **(A)** The expression levels of *GmGOLS*. **(B)** The expression levels of *GmP5CS*. **(C)** The expression levels of *GmNCED1*. **(D)** The expression levels of *GmDREB1*. Values in the figure represent the means ± SD of three technical replicates. Student’s *t*-test for pair comparison in each treatment: **P < 0.01.

We also checked the expression of genes associated with the drought stress response. The expression levels of *GmGOLS*, *GmP5CS*, *GmNCED1*, and *GmDREB1* genes of GO-treated plants were not significantly different from WT before the drought. After 12 days of drought treatment, *GmGOLS*, *GmP5CS*, *GmNCED1*, and *GmDREB1* genes in GO-treated soybeans were significantly increased at 91% ± 0.15, 118% ± 0.26, 64% ± 0.12, and 160% ± 0.35 higher, respectively, than in the WT under natural drought stress (Figure 10). After 12 days of PEG treatment, the expression of *GmGOLS*, *GmP5CS*, *GmNCED1*, and *GmDREB1* genes in GO-treated soybeans was significantly higher by 278% ± 0.65, 143% ± 0.41, 104% ± 0.35, and 154% ± 0.27, respectively, than in the PEG-treated soybeans under simulated drought stress (Figure 10). Our results showed that GO treatment significantly increased the expression of drought-resistant genes including *GmGOLS*, *GmP5CS*, *GmNCED1*, and *GmDREB1* in soybeans, thus improving the plants’ drought tolerance.

## Discussion

Drought is the most important abiotic stress that limits soybean yield (Radhakrishnan and Lee, 2013). Drought tolerance and improvement of soybean drought tolerance have been promising topics for scientific research. Graphene oxide (GO), a novel nanomaterial as a soil water retention agent, plays a role in the growth and development of plants (Shen et al., 2018; Xie et al., 2020). GO not only increased the drought tolerance of in *Zea mays* L. and *Paeonia ostii* (Zhao et al., 2020; Lopes et al., 2021), but also enhanced the saline-alkali tolerance of *Medicago sativa* L. (Chen et al., 2021). In addition, the previous studies showed that the surface morphology and internal structure of *Paeonia ostii* roots were not obviously different between GO and without GO treatment using SEM and TEM. Raman spectroscopy detection also indicated the absence of GO in the cells of the *Paeonia ostii* roots (Zhao et al., 2020). He et al. (2018a) also confirmed that GO could not accumulate in *Spinacia oleracea* L. seedlings cultivated in the soil. Which demonstrated that GO application not accumulate in plant tissue. In this study, we analyzed the effects of GO on the drought tolerance of soybeans. Our results showed that GO improves the mechanism of plant drought tolerance in terms of water content, root growth state, membrane stability, stomatal control, plant hormones, and gene expression.

Drought stress inhibits root elongation and lateral root formation, limiting the uptake of nutrients from the soil because of significant reductions in total root length, surface area, and volume (Sun et al., 2012). Rehman (2014) reported that a larger or more efficient root system determines plant tolerance to drought stress. Soybeans appear to adapt to adverse conditions by producing longer roots that take up more available water (Hoogenboom et al., 1987). Our findings show that GO treatment greatly increased soybean growth and root parameters, including total root length, average root diameter, total root surface area, and total root volume. Therefore, GO can improve root water absorption ability and increase the water content of soybean plants. Similar observations have been reported in *Oryza sativa* (Kondo et al., 2003), *Cicer arietinum* (Kashiwagi et al., 2006), and *Arachis hypogaea* Linn. (Jongrungklang et al., 2011).

The RWC values in the GO-treated leaves are considerably greater than in the non-GO-treated leaves under both types of drought stress conditions. That is evidence that the GO-treated soybeans absorbed water efficiently and prevented water loss. Similar results have been previously reported (Chaparzadeh and Mehrnejad, 2013). Pro is accumulated in several species in response to drought (Molla et al., 2014). However, in soybeans already exposed to GO, drought did not appear to further increase osmotic stress, since proline levels did not increase. Thus, it appears that pre-exposure to GO induced biochemical changes that pre-adapted roots to the osmotic effects of drought, and cells evidenced lower adjustments when effectively exposed to drought conditions, i.e., pre-exposure to GO seemed to induce systemic tolerance to drought in soybeans. The induction of systemic drought tolerance was already reported by Cho et al. (2008) in *Arabidopsis thaliana* plants exposed to 2,3-butanediol. Our study showed that this effect may also be induced by GO nanosheets. Induction of the cellular antioxidant machinery is critical for protecting plants against the adverse effects of abiotic stresses (Hameed et al., 2011). This may be due to a more efficient antioxidative system (Sharma et al., 2012). Our results showed that SOD, POD, CAT, and APX activities were significantly increased in stressed GO-treated plants, whereas the levels of H_2_O_2_ and MDA were lower. Furthermore, we discovered a negative correlation between SOD/POD/CAT/APX activities and MDA/H_2_O_2_ concentrations. These findings suggested that the antioxidant defense machinery remained operative throughout the stress period, enabling plants to adapt to such conditions (also see work by Pérez-Clemente et al., 2013). Wu et al. (2016) reported that stimulating the activity of antioxidant enzymes can remove excess reactive oxygen species to a certain extent, thereby reducing oxidative stress damage. Taken together, our findings showed that GO could increase the activity of the antioxidant system and reduce the accumulation of MDA and H_2_O_2_, thereby reducing oxidative stress damage in soybeans.

The chlorophyll content is one of the important indexes to measure the photosynthetic capacity of plants (Pan et al., 2008). SPAD values can reflect the relative content of chlorophyll in leaves. In this study, SPAD values decreased with the development of drought stress, which is consistent with previous studies (Xie et al., 2014). The GO treatment increased the relative chlorophyll concentration and boosted photosynthetic intensity, allowing soybeans to withstand drought stress. The non-photochemical energy dissipation by PSII antenna pigments often leads to the reduction in Fo. However, damage or reversible inactivation of PSII reaction centers can decrease Fo and Fv/Fm (Björkman and Demmig, 1987). In our study, the Fo of soybean leaves increased during drought stress, indicating that the energy received by the PSII antenna pigments was not transported to the photochemical processes. The energy was lost as fluorescence and heat dissipation when photoprotective systems were activated. The heat dissipation of the PSII reactions can be evaluated by monitoring the changes in qN. To prevent damaging PSII reaction center, the extra energy was effectively dissipated by increasing qN (Golding and Johnson, 2003; Salam et al., 2011). Our findings showed that the qN in GO-treated leaves increased significantly under drought stress, implying that the excess energy in the leaves was dissipated as heat. It effectively reduces damage to the photosynthetic organs or avoids photochemical inhibition due to decreased openness in the reaction centers. This was evident from an increase in Fo, a decrease in qP, and greater values of Fv/Fm during the later stage of drought stress.

The anatomical structure of roots undergoes a series of changes in response to various adverse environments (Wang et al., 2015). Plants can improve their water transport rate by lowering cortex thickness (Zhang et al., 2019) and increasing the ratio of xylem to the phloem (Zhao et al., 2011; Chen et al., 2017) so that they can better adapt to adversity. Our results showed that the cortex of the GO-treated root was significantly thinner than non-Go-treated roots. GO helps the plant adapt to drought by reducing the thickness of the root cortex. The GO treatment significantly increased the thickness of xylem and phloem in soybean roots, ensuring that GO-treated roots could better transport water and absorb nutrients under drought stress. Studies have shown that a thicker upper epidermis of leaves can prevent water loss, reduce transpiration, and enhance the water retention capacity of plants (Zhang et al., 2017). Tang et al. (2014) and Guo and Wu (2018) have shown that the thicker palisade tissue has a larger amount of water and chloroplast storage, which improves photosynthetic efficiency and thus drought tolerance. Our findings revealed that GO adapts to drought by increasing the thickness of the palisade tissue.

Plant defense is regulated by phytohormones such as ABA, SA, JA, and IAA, which create a network of signal transduction pathways that lead to a cascade of events that allow plants to adjust physiologically to a variety of stressors (Pieterse et al., 2009). In our study, ABA content in GO-treated soybeans increased under both drought and PEG stresses, following the results of previous studies (Aimar et al., 2011). Under various abiotic stresses, SA can help plants acclimate and enhance their degree of tolerance by improving their antioxidant capacity (Yang et al., 2004). We also found that JA content in the GO-treated leaves was higher than in the non-GO-treated leaves. Drought stress decreases IAA concentration in plants, which slows their development (Kang et al., 2020). Studies have shown that IAA enhances the activity of reservoir organs and directional induction of assimilate transport (Lenoble et al., 2003). In our study, drought stress greatly decreased the IAA content in soybean leaves, but GO treatment significantly alleviated the decline of IAA content. Taken together, these findings showed that GO promotes soybean drought adaptation by regulating hormone content.

The δ-1-pyrroline-5-carboxylate synthetase (*P5CS*) synthase is the key rate-limiting enzyme for Pro synthesis. The *P5CS* gene expression is linked to Pro accumulation during osmotic stress in *Arabidopsis thaliana*, *Opuntia ficus*-*indica*, and *Brassica napus* (Kubala et al., 2015). In our study, GO treatment increased the expression of the soybean *GmP5CS* gene and improved the accumulation of Pro to achieve drought tolerance under both natural and simulated drought stress. The dehydration-responsive element-binding (*DREB1*) protein binds to the promoter of genes such as responsive dehydration 29A (*RD29A*), promoting their expression in response to drought, salinity, or low temperature (Gao et al., 2007). Our study found that drought increases the expression level of *GmDREB1*. However, the expression level of *GmDREB1* in GO-treated soybeans was significantly higher than in non-GO-treated soybeans. The 9-cis-epoxycarotenoid dioxygenases (*NCED*) catalyze the rate-limiting step in ABA biosynthesis. The expression of *NCED1* gene induces and determines the amount of ABA accumulation (Yamaguchi-Shinozaki and Shinozaki, 2005). The ABA signal also affects the level of drought tolerance in soybeans (Radhakrishnan and Lee, 2013). Our results showed that the expression of the *GmNCED1* gene and the ABA content of GO-treated soybeans were significantly higher than those not treated with GO under both natural and simulated drought stress. Therefore, GO promoted the expression of *GmP5CS*, *GmGOLS*, *GmDREB1*, and *GmNCED1* genes, resulted in the drought tolerance of soybean.

Our findings showed that GO application has potential in improving soybean plant tolerance to drought stress and elucidated the mechanisms for drought tolerance with enzyme activity assays and gene expression quantification, as well as anatomic structure analysis of roots and leaves after drought stress. However, whether its biological effect will be negative with the accumulation of GO remains to be further studied in soybean. At the same time, the existing research lacks the molecular mechanism analysis of graphene’s influence on plant stress tolerance. Therefore, in the future, we propose to analyze the interaction mechanism between graphene and plants by combining multiple groups of omics data and molecular biology methods.

## Data Availability Statement

The datasets presented in this study can be found in online repositories. The names of the repository/repositories and accession number(s) can be found in the article/Supplementary Material.

## Author Contributions

JL and JC contributed to the conception of the study. LZ performed the experiment. XF and AL contributed significantly to analysis and manuscript preparation. LZ performed the data analyses and wrote the manuscript. JL and WW helped perform the analysis with constructive discussions. All authors listed have made a substantial, direct, and intellectual contribution to the work, and approved it for publication.

## Conflict of Interest

The authors declare that the research was conducted in the absence of any commercial or financial relationships that could be construed as a potential conflict of interest.

## Publisher’s Note

All claims expressed in this article are solely those of the authors and do not necessarily represent those of their affiliated organizations, or those of the publisher, the editors and the reviewers. Any product that may be evaluated in this article, or claim that may be made by its manufacturer, is not guaranteed or endorsed by the publisher.
