# Supplementary material for: Graphene Oxide, a Novel Nanomaterial as Soil Water Retention Agent, Dramatically Enhances Drought Stress Tolerance in Soybean Plants
Source: Front Plant Sci. 2022 Feb 15;13:810905. doi: 10.3389/fpls.2022.810905 (PMC8886204; doi:10.3389/fpls.2022.810905)
Supplement: Supplementary file 1 [file Table_1.DOCX]

| Gene | Forward-primer(5’-3’) | Reverse-primer(5’-3’) |
| --- | --- | --- |
| *GmGOLS* | CCCAAATCCTACTCTTGTGACC | GGAGCCATGATCTCAGTGATG |
| *GmP5CS* | TGCAGAGGGGCTGATTTTGG | CTAGTGCATCAGGGCGAGAC |
| *GmNCED1* | TTCTTCCAAATGGTGTCGACG | GGCATTGACAATCTGCAGCTC |
| *GmDREB1* | ACCATGGAAGACAGGGATCACT | CGCGGTGTTGATTCTTGAGC |
| *GmActin* | CTTCCCTCAGCACCTTCCAA | GGTCCAGCTTTCACACTCCAT |

Table S1 Primers used in fluorescence quantitative PCR experiments
