# Supplementary material for: Graphene Oxide, a Novel Nanomaterial as Soil Water Retention Agent, Dramatically Enhances Drought Stress Tolerance in Soybean Plants
Source: Front Plant Sci. 2022 Feb 15;13:810905. doi: 10.3389/fpls.2022.810905 (PMC8886204; doi:10.3389/fpls.2022.810905)
Supplement: Supplementary file 2 [file Data_Sheet_1.docx]

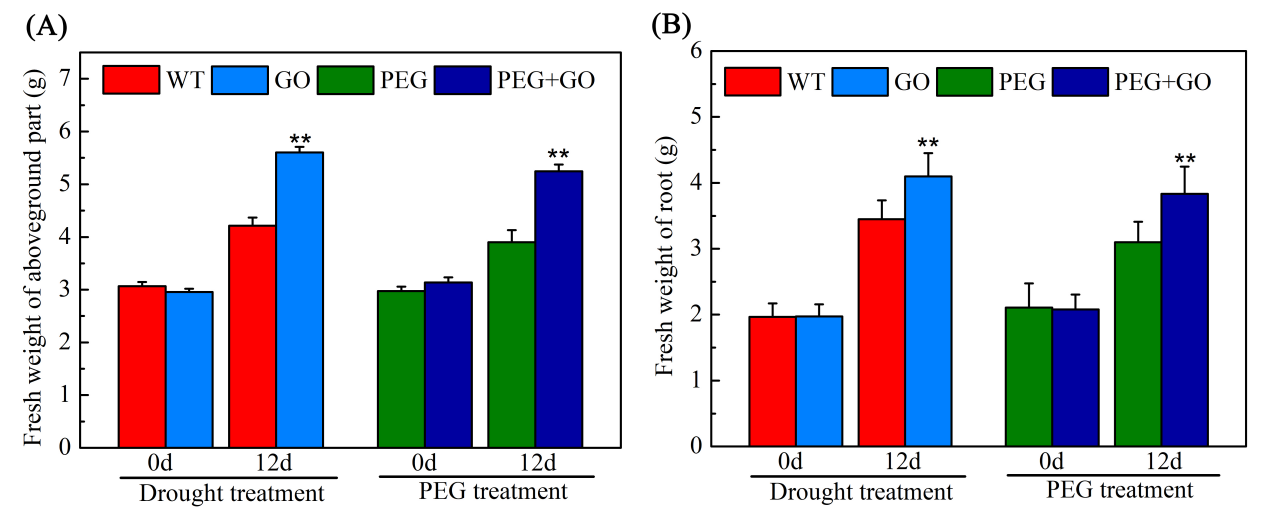


**Supplementary Figure 1.** Effects of GO on fresh weight of soybean under drought and PEG stress. (A) Fresh weight of aboveground parts. (B) Fresh weight of roots. Values in the figure represent the means±SD of three replicates. Student’s t-test for pair comparison in each treatment: ***P* < 0.01.


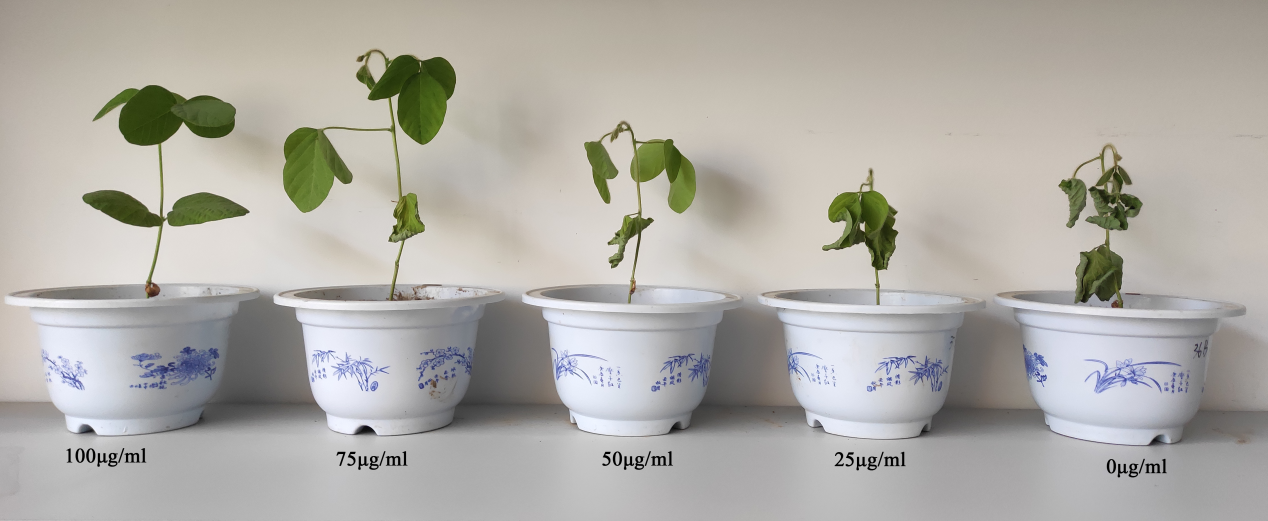


**Supplementary Figure 2.** Phenotypic characteristics of drought resistance of soybean treated with different concentrations of GO.
